# Phenotypic characterisation of *Pasteurella multocida* strains isolated from rabbits in Poland

**DOI:** 10.2478/jvetres-2026-0029

**Published:** 2026-06-05

**Authors:** Sylwia Budniak, Agnieszka Kędrak-Jabłońska, Krzysztof Szulowski

**Affiliations:** Department of Bacteriology and Bacterial Animal Diseases, National Veterinary Research Institute, 24-100 Puławy, Poland

**Keywords:** *Pasteurella multocida*, rabbit, biochemical profile, capsular and somatic serotyping

## Abstract

**Introduction:**

*Pasteurella multocida* is an important pathogen in many species of domestic and wild mammals, as well as birds. It is also a commensal in the nasopharynx of clinically healthy animals. The aim of the study was to characterise and classify field strains of *P. multocida* isolated from rabbits in Poland.

**Material and Methods:**

A total of 115 field strains isolated from rabbits in 1999–2020 were used. The examined strains were evaluated morphologically and microscopically, and their ability to produce catalase and oxidase was determined. Identification of *P. multocida* strains was performed on basis of biochemical characteristics and serotype classification.

**Results:**

Based on the biochemical profile assessment using API 20E and the ID 32E (bioMérieux), the strains isolated from rabbits were identified as *P. multocida*. Among 17.4% were classified as *P. multocida* subsp. *multocida*, 80% as ornithine decarboxylase negative *P. multocida* subsp. *multocida* and 2.6% as *P. multocida* subsp. *septica*. The capsule of type A was found in 87.8% of strains, type D in 8.7%, and type F in 3.5%. The somatic antigen of serotype 12 was present in 64.3% of strains, and serotype 3 in 36.7% of strains. The capsular type A was predominant among strains from rabbits that were asymptomatic carriers, while the capsular type D and F were found mainly in strains isolated from sick animals. In turn, the somatic antigen of serotype 12 was present in strains from both healthy and diseased animals, while strains of serotype 3 were much more frequently isolated from diseased rabbits.

**Conclusion:**

The conducted research allowed to classify and obtain new in-depth data on the population structure of *P. multocida* strains isolated from rabbits in Poland.

## Introduction

The *Pasteurella* genus belongs to the Pasteurellaceae family, which comprises a large and diverse group of Gram-negative microorganisms (8). Pasteurellaceae Pohl 1981 is classified within the Proteobacteria phylum, Gammaproteobacteria class and Pasteurellales order, and currently consists of 17 genera and over 60 species of microorganisms. These have been isolated from animals and humans (11). Comparative genomic and phylogenetic analyses of Pasteurellaceae have revealed that many members of this highly diverse family have been insufficiently classified. Indeed, numerous species within the Pasteurellaceae family have already been reclassified, including *Mannheimia haemolytica, Bibersteinia trehalosi, Actinobacillus ureae, Avibacterium gallinarum, Avibacterium volantium, Avibacterium avium* and *Gallibacterium anatis* (8, 11). Advances in microbiological diagnostics mean that the taxonomy of the Pasteurellaceae family continues to undergo significant changes and revisions.

*Pasteurella multocida* is a commensal or opportunistic pathogen found in the upper respiratory tract of most domestic and wild animals. This bacterium has been isolated from chickens, turkeys, wild birds, cattle and American bison, pigs, rabbits, dogs, domestic cats and large wild felids such as tigers, leopards, cougars and lions, as well as from chimpanzees, marine mammals including seals, sea lions and walruses, and even from Komodo dragons (15, 31). It was first described by Italian botanist Vittore Trevisan in 1887. He proposed the genus name *Pasteurella* in honour of the famous French microbiologist and chemist Louis Pasteur. The researcher first isolated the bacterium in 1880 as the causative agent of fowl cholera. He conducted several experiments infecting chickens to understand the mechanism of transmission of the microorganism and assess its pathogenicity (35). In turn, Mutters *et al*. (24) reclassified the *Pasteurella* genus based on DNA–DNA hybridisation studies. They demonstrated that *Pasteurella sensu stricto* comprises at least eleven species, including *P. multocida, P. dagmatis, P. canis, P. stomatitis* and *Pasteurella* species B. Within *P. multocida*, three subspecies have been identified: *P. multocida* subsp. *multocida, P. multocida* subsp. *septica* and *P. multocida* subsp. *gallicida*, which show 84% to 100% DNA relatedness, despite their predilection for different hosts and the diversity of the clinical symptoms they cause. Research by Petersen *et al*. (29), based on a comparison of the 16S rRNA and *atpD* gene sequences, confirmed the genetic homogeneity of *P. multocida*. Similarly, research by Kuhnert *et al*. (18) demonstrated that the subspecies of *P. multocida*, corresponding to distinct phenotypic variants, exhibit at least 98.5% similarity in their 16S rRNA sequences.

In 2002, Capitini *et al*. (3) isolated a *Pasteurella* strain from a girl who had been bitten by a tiger. Based on the phenotypic characteristics of this strain and comparisons of 16S rRNA gene sequences, it was regarded as a putative new subspecies, and the name *P. multocida* subsp. *tigris* was proposed. This strain possessed characteristics typical of *P. multocida* but did not ferment sucrose and mannitol (24). However, subsequent studies by Christensen *et al*. (9), including DNA–DNA hybridisation, recommended this strain be reclassified as a new taxon, Bisgaard taxon 45. Although it is genotypically related to *P. multocida*, its polyamine pattern differs from those of all *P. multocida* subspecies. Separate taxa within the *Pasteurellaceae* family, such as Bisgaard taxon 45, Bisgaard taxon 46 and *Pasteurella* species B, include strains the final systematic positions of which have not yet been conclusively determined.

The aim of this study was to characterise and classify *P. multocida* strains isolated from rabbits in Poland. The biochemical properties and serotype classification based on the identification of capsular and somatic antigens of domestic *P. multocida* strains, were determined.

## Material and Methods

### Bacterial strains

The study used 115 field strains isolated between 1999 and 2020 in Poland. The strains came from rabbits from both large-scale farms and individual small farms. The large-scale farms were located in Chorzelów in the Podkarpackie voivodeship, Gdańsk in the Pomorskie voivodeship, Małogoszcz in the Świętokrzyskie voivodeship and Zduny in the Wielkopolskie voivodeship. The individual small farms came from various areas of Poland. The first group consisted of strains isolated from nasal swabs of rabbits with respiratory symptoms or being asymptomatic carriers. This group comprised 87 strains, of which 73 were isolated from rabbits with symptoms of rhinitis, and the remaining 14 came from clinically healthy animals. The second group consisted of 28 strains from dead rabbits. Twenty-seven strains were isolated from internal organs: 26 from the lungs and one strain from the heart, while one strain came from an abscess on the skin. In addition, reference strains of *P. multocida* were used as controls: P8 and P1059 of *P. multocida* type A and Kobe 6 and P27 of type D obtained from Dr Namioka of the National Institute of Animal Health, Tokyo, Japan, B850 and P932 of type B from the Institut d’Élevage et de Médecine Vétérinaire des Pays Tropicaux, Paris, France and P4218 and P3695 of type F from Dr Rimler of the National Animal Disease Center, Ames, IA, USA. The reference strains X73, M1404, P1059, P1662, P1702, P2192, P1997, P1581, P2095, P2100, P903, P1573, P1591, P2225, P2237 and P2723 originated from the National Animal Disease Center, Ames, IA, USA.

### Morphological and microscopic examination

The strains were cultured on 5% horse blood agar, MacConkey agar, dextrose-starch agar (DSA) and nutrient broth. After incubation for 24 h at 37°C, the appearance and size of colonies on solid media were assessed, as well as the turbidity and sediment formation in the liquid medium. Microscopic examination was then performed on preparations stained using the Gram method, modified by Kopeloff. Gram-negative rods were subjected to further analysis.

### Catalase test

A colony taken from a bacterial culture grown on solid medium was suspended in a drop of 3% hydrogen peroxide solution on a microscope slide. The release of gas bubbles indicated a positive reaction.

### Cytochrome oxidase activity test

An OXITEST commercial oxidase test (Erba Lachema, Brno, Czech Republic) was used according to the manufacturer’s instructions. A colour change of the filter paper strip to dark blue indicated the presence of oxidase-positive bacteria.

### Biochemical profile assessment

The biochemical profile was determined using API 20E and ID 32E tests (bioMérieux, Marcy-l’Étoile, France), performed in accordance with the manufacturer’s instructions. The strains were also inoculated into liquid media for fermentation of dulcitol, trehalose, xylose and maltose. Incubation was carried out at 37°C for 6 d. A colour change from blue to yellow indicated a positive reaction resulting from sugar fermentation.

### Determination of capsular type by the indirect haemagglutination test (IHA)

The indirect haemagglutination test was performed by the micromethod according to Carter (4, 5). Each strain was tested with reference anticapsular sera of types A, B, D and F, produced in previous years at the Department of Bacteriology and Bacterial Animal Diseases, National Veterinary Research Institute in Puławy, Poland. Serum dilutions ranging from 1:10 to 1:2,560 were used in the assays. The microplates were left for 2 h at 21°C. after which the first reading was taken. The plates were then incubated for a further 18 h at 5°C, and the second reading was performed. A positive result was indicated by the presence of haemagglutination at the bottom of the well.

### Determination of the somatic antigens by the agar gel immunodiffusion test (AGID)

The assay was performed following the method of Heddleston *et al*. (16), with modifications introduced by other authors (32, 36). Sera positive for 16 Heddleston reference strains obtained in previous years by the Department of Bacteriology and Animal Bacterial Diseases, National Veterinary Research Institute in Puławy, Poland, were used for the tests. The specificity of all sera was verified against antigens representing the 16 Heddleston serotypes. The plates were placed in a humid chamber and incubated for 24 h at 37°C and for an additional 24 h at 21°C. The results were read after 48 h. A positive reaction was indicated by the formation of a precipitation line.

## Results

**Fig. 1A and B:**
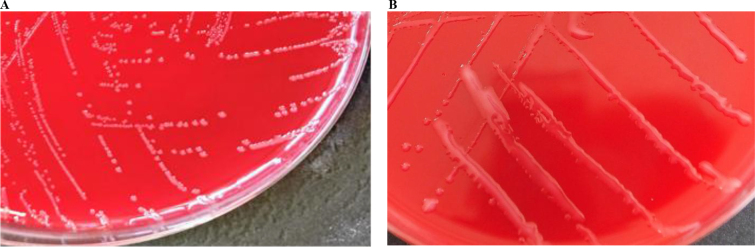
Growth of *Pasteurella multocida* colonies on 5% horse blood agar

For all tested strains grown on 5% horse blood agar, growth was observed in the form of smooth, small, semitransparent greyish colonies with a diameter of 1–3 mm and regular edges (Fig. 1A), as well as larger, mucoid colonies measuring 3–4 mm in diameter (Fig. 1B). No haemolysis was detected in any of the strains. When cultured in nutrient broth, the strains grew as a homogenous turbid suspension. On DSA medium, the strains formed smooth, yellow, opalescent colonies measuring 2–3 mm in diameter, while no growth was observed on MacConkey agar. In microscopic preparations stained by the Gram method, all the strains appeared as short, slender, Gram-negative rods. All bacterial strains tested were shown to produce both catalase and oxidase. The results of the biochemical profile assessment using the API 20E and ID 32E tests are presented in Tables 1 and 2. Based on the results of both tests, the strains isolated from rabbits were identified as *P. multocida*.

**Table 1.** Biochemical profile assessment of *Pasteurella multocida* strains using the API 20E

Reaction/enzyme	Number of strains showing positive reactions (%)		
	*P. multocida* subsp. *multocida*	*P. multocida* subsp. *multocida* ornithine-negative	*P. multocida* subsp. *septica*
β-galactosidase	0	0	0
Arginine dihydrolase	0	0	0
Lysine decarboxylase	0	0	0
Ornithine decarboxylase	20(100)	0	3 (100)
Citrate utilisation	0	0	0
Hydrogen sulphide production	0	0	0
Urease	0	0	0
Tryptophan deaminase	0	0	0
Indole production	17(85)	92 (100)	1 (33)
Acetoin production	0	0	0
Gelatinase	0	0	0
D-glucose	20 (100)	92 (100)	3 (100)
D-mannitol	20 (100)	92 (100)	3 (100)
Inositol	0	0	0
D-sorbitol	20 (100)	92 (100)	0
L-rhamnose	0	0	0
D-saccharose	20 (100)	92 (100)	3 (100)
D-melibiose	0	0	0
Amygdalin	0	0	0
L-arabinose	0	0	0
Cytochrome oxidase	20 (100)	92 (100)	3 (100)
Nitrate reduction	20 (100)	92 (100)	3 (100)
Total	20	92	3

**Table 2.** Biochemical profile assessment of *Pasteurella multocida* strains using the ID 32E

Reaction/enzyme	Number of strains showing positive reactions (%)		
	*P. multocida* subsp. *multocida*	*P. multocida* subsp. *multocida* ornithine-negative	*P. multocida* subsp. *septica*
Ornithine decarboxylase	20(100)	0	3 (100)
Arginine dihydrolase	0	0	0
Lysine decarboxylase	0	0	0
Urease	0	0	0
L-arabitol	0	0	0
Galacturonate	0	0	0
5-ketogluconate	0	0	0
Lipase	0	0	0
Phenol red	0	0	0
β-glucosidase	0	0	0
Mannitol	20 (100)	92(100)	3 (100)
Maltose	0	0	0
Adonitol	0	0	0
Palatinose	0	0	0
β-glucuronidase	0	0	0
Malonate	0	0	0
Indole production	17 (85)	92 (100)	1 (33)
N-acetyl-β-glucosaminidase	0	0	0
β-galactosidase	0	0	0
Glucose	20 (100)	92 (100)	3 (100)
Sucrose	20 (100)	92 (100)	3 (100)
L-arabinose	0	0	0
D-arabitol	0	0	0
α-glucosidase	0	0	0
α-galactosidase	0	0	0
Trehalose	7 (35)	0	0
Rhamnose	0	0	0
Inositol	0	0	0
Cellobiose	0	0	0
Sorbitol	20 (100)	92 (100)	0
α-maltosidase	0	0	0
L-aspartic acid arylamidase	0	0	0
Total	20	92	3

The API 20E test showed that all *P. multocida* strains produced cytochrome oxidase; fermented glucose, mannitol and sucrose; and reduced nitrates to nitrites. Ornithine decarboxylase was produced by 20% of the strains, while 95.6% produced indole and 97.4% fermented sorbitol. None of the strains utilised citrate; produced galactosidase, arginine dihydrolase, lysine decarboxylase, hydrogen sulphide, urease, tryptophan deaminase, acetoin or gelatinase; nor fermented inositol, rhamnose, melibiose, amygdalin or arabinose (Table 1).

The ID 32E test results confirmed the production of ornithine decarboxylase and indole, as well as sorbitol fermentation, in the same proportion of strains. All *P. multocida* strains fermented mannitol, glucose and sucrose. Trehalose was fermented by 6.1% of the tested strains. It was found that the strains did not produce arginine dihydrolase, lysine decarboxylase, urease, lipase, β-glucosidase, β-glucuronidase, malonate, N-acetyl-β-glucosaminidase, β-galactosidase, α-glucosidase, α-maltosidase or L-aspartic acid arylamidase. All strains also failed to ferment D-arabitol, galacturonate, 5-ketogluconate, phenol red, adonitol, palatinose, L-arabinose, rhamnose and inositol (Table 2).

The ID 32E test results confirmed the production of ornithine decarboxylase and indole, as well as sorbitol fermentation, in the same proportion of strains. All *P. multocida* strains fermented mannitol, glucose and sucrose. Trehalose was fermented by 6.1% of the tested strains. It was found that the strains did not produce arginine dihydrolase, lysine decarboxylase, urease, lipase, β-glucosidase, β-glucuronidase, malonate, N-acetyl-β-glucosaminidase, β-galactosidase, α-glucosidase, α-maltosidase or L-aspartic acid arylamidase. All strains also failed to ferment D-arabitol, galacturonate, 5-ketogluconate, phenol red, adonitol, palatinose, L-arabinose, rhamnose and inositol (Table 2).

The results of sugar fermentation tests showed that none of the strains fermented dulcitol or maltose. Trehalose was fermented by 6.1% of all strains tested, and xylose by 97.4%. The results of the sugar fermentation tests are presented in Table 3.

**Table 3.** Sugar fermentation of *Pasteurella multocida* strains isolated from rabbits

Sugar	Number of strains showing positive reactions (%)		
	*P. multocida* subsp. *multocida*	*P. multocida* subsp. *multocida* ornithinenegative	*P. multocida* subsp. *septica*
Dulcitol	0	0	0
Trehalose	7 (35)	0	0
Xylose	17(85)	92 (100)	3 (100)
Maltose	0	0	0
Total	20	92	3

All strains isolated from rabbits were classified according to the criteria of Mutters *et al*. (24, 25) and Bisgaard et *al*. (1). A proportion equal to 17.4% of the isolates was identified as *P. multocida* subsp. *multocida*, and 80% as ornithine decarboxylase negative *P. multocida* subsp. *multocida*. The remaining 2.6% of the strains were classified as *P. multocida* subsp. *septica*.

The study showed that 20% of all strains produced ornithine decarboxylase. These included all strains which were *P. multocida* subsp. *multocida* and *P. multocida* subsp. *septica*. Indole production was observed in 100% of ornithine decarboxylase negative *P. multocida* subsp. *multocida* strains, 85% of *P. multocida* subsp. *multocida* strains and 33% of *P. multocida* subsp. *septica* strains. All strains of *P. multocida* subsp. *multocida* and ornithine decarboxylase negative *P. multocida* subsp. *multocida* fermented sorbitol, whereas none of the *P. multocida* subsp. *septica* strains did. Among the 20 *P. multocida* subsp. *multocida* strains, 35% fermented trehalose and 85% fermented xylose. In contrast, all ornithine decarboxylase negative *P. multocida* subsp. *multocida* and *P. multocida* subsp. *septica* strains failed to ferment trehalose but did ferment xylose.

Among the strains obtained from clinically healthy rabbits, 7.1% were *P. multocida* subsp. *multocida*, while 92.9% were ornithine decarboxylase negative *P. multocida* subsp. *multocida*. The strains from rabbits showing symptoms of rhinitis were *P. multocida* subsp. *multocida* in a 16.4% proportion, and 83.6% were ornithine decarboxylase negative *P. multocida* subsp. *multocida*. Among the strains obtained from dead animals, 25% were identified as *P. multocida* subsp. *multocida*, 64.3% as ornithine decarboxylase negative *P. multocida* subsp. *multocida* and 10.7% as *P. multocida* subsp. *septica*.

In the next stage, the capsular antigens were determined using IHA (4, 5). It was found that the capsular antigen of type A was present in 87.8% of rabbits, type D in 8.7% and type F in 3.5% of animals (Table 4). Strains possessing capsule of type A resolved all biochemical groups. Of them, 14.8% were *P. multocida* subsp. *multocida*, 84.2% ornithine decarboxylase negative *P. multocida* subsp. *multocida* and the remaining 1% were *P. multocida* subsp. *septica*. In the group of strains with capsular type D, 30% were classified as *P. multocida* subsp. *multocida* and 70% as ornithine decarboxylase negative *P. multocida* subsp. *multocida*. Among the strains with capsular type F, 50% were identified as *P. multocida* subsp. *multocida*, and 50% as *P. multocida* subsp. *septica*.

**Table 4.** Distribution of *Pasteurella multocida* capsular antigens

		Somatic antigen number of strains (%)		Number of strains (%)
		3	12	
Capsular antigen	A	38 (37.6)	63 (62.4)	101 (87.8)
	D	2 (20.0)	8 (80.0)	10 (8.7)
	F	1 (25.0)	3 (75.0)	4 (3.5)
Total		41	74	115

In the next stage, the capsular antigens were determined using IHA (4, 5). It was found that the capsular antigen of type A was present in 87.8% of rabbits, type D in 8.7% and type F in 3.5% of animals (Table 4). Strains possessing capsule of type A resolved all biochemical groups. Of them, 14.8% were *P. multocida* subsp. *multocida*, 84.2% ornithine decarboxylase negative *P. multocida* subsp. *multocida* and the remaining 1% were *P. multocida* subsp. *septica*. In the group of strains with capsular type D, 30% were classified as *P. multocida* subsp. *multocida* and 70% as ornithine decarboxylase negative *P. multocida* subsp. *multocida*. Among the strains with capsular type F, 50% were identified as *P. multocida* subsp. *multocida*, and 50% as *P. multocida* subsp. *septica*.

Strains isolated from both clinically healthy rabbits and those with symptoms of rhinitis belonged mainly to capsular type A. In the group of animals with symptoms of rhinitis, only 4.1% of strains had capsular antigen D. Capsule of type A also dominated among strains isolated from dead rabbits (60.7%), while type D comprised 25.0% and type F 14.3%.

Next, somatic antigens were detected using AGID (16). It was found that 35.7% of the tested strains were serotype 3, while 64.3% were classified as serotype 12 (Table 5). In addition to the clearly marked main band resulting for every one of the 41 serotype 3 strains, 28 strains showed weaker precipitation lines with sera specific for serotypes 1, 2, 5, 10 and 12. In single strains, a weak band with sera of serotypes 11 and 16 was also present. In turn, 23 serotype 12 strains showed only a clearly marked main precipitation band, while the remaining 51 strains of this serotype also showed faint precipitation lines with sera specific for one or more of serotypes 1, 2, 3, 4, 5, 6, 8, 10, 11, 14 and 16.

It emerged that 37.6% of strains in capsular antigen type A were Heddleston somatic antigen serotype 3, and 62.4% were antigen serotype 12. Among the strains with capsular antigen type D, 20% belonged to serotype 3 and 80% to serotype 12. In capsular type F, 25% of the strains had somatic antigen 3 and 75% had antigen 12 (Table 4).

**Table 5.** Distribution of *Pasteurella multocida* somatic antigens

		Capsular antigen numer of strains (%)			Number of strains (%)
		A	D	F	
Somatic antigen	3	38 (92.7)	2 (4.9)	1 (2.4)	41 (35.7)
	12	63 (85.1)	8 (10.8)	3 (4.1)	74 (64.3)
Total		101	10	4	115

The vast majority of *P. multocida* strains (92.7%) of serotype 3 possessed capsular antigen type A, while 4.9% had antigen type D and 2.4% F. A large majority (85.1%) of serotype 12 strains belonged to capsular type A, while 10.8% type D and 4.1% had type F (Table 5).

All strains isolated from clinically healthy animals belonged to serotype 12. Among strains from rabbits with symptoms of rhinitis, 63% belonged to serotype 12 and 37% to serotype 3. In turn, strains obtained from dead animals resolved to each serotype in 50% proportions.

## Discussion

*Pasteurella multocida* is a Gram-negative bacterium found in many different species of domestic and wild animals, including primates, and humans. These microorganisms can be present as a commensal on mucous membranes, mainly in the respiratory system of vertebrates, or occur as primary or opportunistic pathogens (34).

The first stage of laboratory diagnosis of *P. multocida* involves classic bacteriological examinations. Preliminary isolation of bacterial cultures for further identification is made possible by the characteristic appearance of colonies on solid media. In our study, the characteristic colony morphology of *P. multocida* was noted. Large, mucoid colonies or small, round, shiny colonies with a greyish tone and regular edges were found on blood agar. Classical diagnostics relying on phenotypic characteristics, such as colony morphology, the ability to fermentation of glucose, mannitol, sucrose, trehalose, dulcitol, and sorbitol, as well as the production of indole and ornithine decarboxylase, are usually sufficient for the identification of *P. multocida* (13, 14). Determining biochemical properties makes it possible to establish certain biochemical profiles of bacterial strains within a given species. This method is used as a preliminary step for further classification of strains based on other techniques (33). However, as reported by Bisgaard *et al*. (1) and Christensen *et al*. (8), *P. multocida* may exhibit atypical biochemical features. Strains negative for mannitol or indole, or those lacking ornithine decarboxylase activity, may occasionally occur.

Many researchers have attempted to classify *P. multocida* biochemically. In studies conducted by Brogden *et al*. (2), all 48 strains isolated from rabbits fermented glucose, sucrose, mannose, mannitol and galactosidase. Most strains also fermented xylose and sorbitol, and 41 strains produced indole. Only two strains fermented lactose and trehalose, and one strain each fermented maltose and dulcitol. Lu *et al*. (20) isolated 42 strains from 135 nasal swabs collected from healthy rabbits, and subjected them to biochemical testing, revealing that 55% of the strains produced indole, while 24% produced ornithine decarboxylase. In our study, almost all strains classified as *P. multocida* fermented xylose, while none fermented arabinose. All strains produced acid from glucose, mannose, sucrose, mannitol and sorbitol. Among the 115 tested strains, 5 were indole-negative, and 92 did not produce ornithine decarboxylase. These results largely match those of Lugo-Marante *et al*. (21), who examined 11 strains isolated from rabbits between 1995 and 2005 and classified them as *P. multocida* subsp. *multocida*. All strains fermented sorbitol, 91% mannitol and all but one xylose. None of the strains fermented maltose, arabinose, dulcitol or trehalose. However, all the strains tested by those authors produced ornithine decarboxylase.

According to Król *et al*. (17), biochemical properties such as the production of acid from sorbitol, trehalose and dulcitol, as well as the production of α-glucosidase, are important for distinguishing between *P. multocida* subsp. *multocida* and *P. multocida* subsp. *septica* strains. Those authors observed that *P. multocida* subsp. *multocida* in most cases produced acid from sorbitol (94.9%) and very rarely produced acid from trehalose (2.6%) or the enzyme α-glucosidase (2.6%). In the present study, all *P. multocida* subsp. *multocida* strains utilised sorbitol, while only 35% produced acid from trehalose, and none produced α-glucosidase. Unlike the findings reported by Król *et al*. (17), for whom *P. multocida* subsp. *septica* fermented sorbitol in 72.4% of strains and trehalose in 65.5%, and produced α-glucosidase in 58.6% of strains, no sorbitol fermentation was observed in the present study among *P. multocida* subsp. *septica* strains.

While biochemical characteristics provide a basis for differentiation of *P. multocida* strains, the analysis of capsular and somatic antigens offers additional insight into their epidemiological distribution and potential association with disease. As early in 1929, Cornelius performed the first serological grouping of *P. multocida* based on the agglutinin absorbance test, resulting in the creation of four groups (10). In 1955, the typing system created by Carter, based on the indirect haemagglutination test, laid the foundations for the current classification of capsular antigens A, B, D and E, and in later years also capsular antigen F (4, 30). In 1972, Heddleston *et al*. (16) developed somatic antigen typing systems using the agar gel immunodiffusion test, which differentiates 16 somatic antigens of *P. multocida*. Currently, the Carter and Heddleston systems for capsular and somatic serotyping are used simultaneously. A complete serotype designation of a *P. multocida* strain consists of the capsular type (according to Carter) followed by the somatic type (according to Heddleston).

Accordingly, capsular and somatic antigen profiles were determined for the 115 strains of this bacterium isolated from rabbits. The capsular antigen frequencies determined were consistent with those reported by other authors from the USA, such as Lu *et al*. (19) and Chengappa *et al*. (7), who found that capsular antigens of types A and D were the most frequently isolated from rabbits. Lu *et al*. (20) reported that 67% of the strains possessed a capsular type A antigen and 5% a capsular type D. According to Chengappa *et al*. (7), among 79 tested *P. multocida* strains from rabbits, 74 had capsular type A, which accounted for as much as 93%, and only 5 strains, that is approximately 6%, had capsular type D. In Italy, capsular type A also predominated among examined strains, occurring in 20 strains out of 39, while types D and F were detected in 9 and 10 strains, respectively (22). Also, Mushin and Schoenbaum (23) classified 94% of the strains which they isolated from rabbits to type A and 6% to type D. Virág *et al*. (38) examined 32 strains obtained from both healthy rabbits and those exhibiting clinical symptoms. They detected the presence of capsular type A in 53% of the strains, type F in 28% and type D in only 9%. The authors concluded that colony morphology, biovar and capsular type showed no correlation with the health status of the rabbits.

In turn, studies on the identification of somatic antigens conducted by Brogden (2), Chengappa *et al*. (7), Lu *et al*. (19) and Percy *et al*. (27) showed that somatic serotype 12 was the dominant one in strains isolated from rabbits. The frequency of its detection ranged from 27% to 93%, while somatic serotype 3 was 5% to 57%. Percy *et al*. (27) found the serotype 12 somatic antigen in 53% of 59 *P. multocida* strains and the serotype 3 in 46%. Brogden (2) showed that over 66% of strains from rabbits were of somatic serotype 12 and 25% were of serotype 3, and observed serotypes 3 and 12, regardless of the place and time of a strain’s isolation. Individual strains were found to express somatic serotypes 1, 4 and 15. Also, as reported by Chengappa *et al*. (7), serotype 12 was the dominant serotype, while others, *i.e*. 1, 3, 4 and 11, were less common. Mushin and Schoenbaum (23) observed that serotype 12 was the main somatic antigen, occurring in 84% of all strains, while antigens 3 and 1 were less common. In the present study, none of the tested strains exhibited somatic serotypes other than 3 and 12. Mushin and Schoenbaum (23) also reported strains that possessed additional faint bands with one or more somatic antigens, such as 5, 7, and 12. In their studies, Chengappa *et al*. (7) and Lu *et al*. (19) found additional faint precipitation lines with sera of different types. Comparably, in addition to a clearly marked main band, we also found weak positive reactions by most strains with sera of other somatic serotypes.

Lu *et al*. (19) noted some interdependence between somatic serotype and strain origin. While they found serotype 12 to be dominant in both healthy and sick rabbits, they isolated serotype 3 from sick rabbits only. We also observed that somatic serotype 12 occurred in both healthy and sick animals, while serotype 3 was isolated much more frequently from sick rabbits. In turn, capsular type A was dominant among asymptomatic carriers, while types D and F were mainly found in sick animals.

Many researchers have attempted to identify relationships between *P. multocida* strains and their virulence. In 1924, Webster first observed that *P. multocida* strains were capable of persisting in the nasal cavity of rabbits. These observations suggested that *P. multocida* strains could differ in their behaviour and in their association with the severity of pasteurellosis (37). This concept was also supported by Okerman *et al*. (26), who concluded from their studies that less virulent strains had capsular type A and caused rhinitis, whereas more virulent strains had capsular type D and were associated with septicaemia.

Studies by other authors have also demonstrated similar relationships between serotypes of strains and clinical forms of the disease. Lu *et al*. (19) attempted to determine the connections between somatic and capsular antigens and various clinical forms of pasteurellosis. They found that both healthy and diseased rabbits were sources of serotypes A:12 and A:3. Similarly, somatic serotype 12 predominated in rabbits with rhinitis as well as in clinically healthy rabbits (12). In turn, the studies conducted by other researchers indicated that somatic serotype 3 was particularly virulent in rabbits, and that serotype A:3 was more virulent than A:12 in both naturally occurring and experimentally induced infections (19, 28). As shown by Percy *et al*. (27), serotypes A:3 and D:3 were associated with acute or purulent pneumonia.

Studies by Carter and Chengappa (6) indicated that serotype A:12 was the most common among *P. multocida* strains obtained from rabbits in the USA. A similar pattern was observed in Poland, the majority of domestic *P. multocida* strains isolated from rabbits were identified as serotype A:12, with A:3 occurring less frequently. The capsular antigen of type D was less prevalent, while the presence of type F was detected in only four strains. Somatic serotype 12 was also predominant within these capsular types.

In this study, phenotypic characteristics were determined, providing new and important information on the population structure of *P. multocida* strains isolated from rabbits in Poland. The conducted research also contributed to improving species identification and serotyping methods for *P. multocida* strains.

## Conclusion

In summary, the analysed strains isolated from rabbits were predominantly classified as ornithine decarboxylase negative *P. multocida* subsp. *multocida* and *P. multocida* subsp. *multocida*, whereas *P. multocida* subsp. *septica* was detected only sporadically. Most strains belonged to capsular type A, while capsular types D and F were observed less frequently. With regard to somatic antigens, serotype 12 was the most commonly identified, whereas serotype 3 occurred more often among strains isolated from sick rabbits. From a practical point of view, these findings constitute baseline data that may be useful for rabbit health protection programmes in Poland and for considerations related to the selection of strains for vaccine preparation. Further research will include the genotypic characterisation of the examined strains.
